# Metabolites from chia seed (*Salvia Hispanica* L.) exert antioxidant effects through activation of the DAF-16 pathway and neuroprotective activity in the *Caenorhabditis elegans* model of Huntington’s disease

**DOI:** 10.1186/s12906-026-05277-7

**Published:** 2026-02-20

**Authors:** Sara Thabit, Reem Hossam El Din, Nermeen El Haddad, Ayat Ajaj, Aya Hesham, Ntakadzeni Edwin Madala, Heba Handoussa

**Affiliations:** 1 Department of Pharmaceutical Biology, Faculty of Pharmacy and Biotechnology, German University in Cairo, New Cairo, 11835 Egypt; 2 Department of Pharmaceutical Microbiology, Faculty of Pharmacy and Biotechnology, German University in Cairo, New Cairo, Egypt; 3 Department of Biochemistry & Microbiology, Faculty of Science, Engineering and Agriculture, University of Venda, P. Bag X5050, Thohoyandou, 0950 South Africa

**Keywords:** Antioxidant, *Caenorhabditis elegans*, DAF-16/FOXO, Huntington’s disease, PolyQ

## Abstract

**Background:**

*Salvia hispanica* L., chia seed, is a well-known highly nutritious nutraceutical normally included in the daily diet of many people worldwide. Despite having a plethora of reported beneficial uses, little is known about its neuroprotective properties, especially in Huntington’s disease (HD). In our study, we focused on testing the antioxidant properties of metabolites from chia (CH) seed and their potential to protect against the development of HD in the in vivo worm model, *Caenorhabditis elegans* (*C. elegans*).

**Methods:**

Using the *C. elegans* model, the oxidative stress resistance capabilities were tested via a survival assay through exposure to a toxic dose of the pro-oxidant juglone. The antioxidant properties were assessed by performing ROS assay to detect the intracellular levels of basal ROS inside the worms. To detect whether the DAF-16/FOXO pathway is involved in the antioxidant activity or not, its activation with its downstream target genes, *sod-3* and *hsp-16.2*, was tested. The neuroprotective activity against several HD-associated phenotypes was evaluated by assessing the number of polyQ150, polyQ35, and polyQ40 aggregation clusters. This study also comprehensively characterized the bioactive secondary metabolites profile of the dichloromethane (DCM) fraction of chia seed using UPLC–q-TOF–ESI–MS/MS.

**Results:**

In our study, CH seed DCM fraction was able to increase the survival of *C. elegans* worms exposed to juglone and could attenuate the basal ROS levels inside the worms. It was also able to activate the DAF-16/FOXO pathway through increasing its nuclear localization, upregulating the expression of SOD-3 and decreasing the expression of HSP-16.2 following juglone exposure. CH seed looks promising as an anti-HD candidate because of its ability to reduce the formation of polyQ35, polyQ40 and polyQ150 clusters. Finally, phytochemical profiling of the active DCM fraction identified seventy five metabolites belonging to various phytochemical classes.

**Conclusions:**

This study shows that CH seed extract decreases oxidative stress and polyQ aggregates accumulation, associated with HD-related phenotypes, in a *Caenorhabditis elegans* model, supporting further investigations.

## Introduction

The antioxidant systems in our bodies balance the levels of reactive oxygen species (ROS) to avoid their abnormal increase that leads to oxidative stress conditions. Oxidative stress is responsible for deleterious effects inside the body, such as damage to cell membranes and alterations in structural and signaling proteins. This leads to misfolding and aggregation of those proteins, causing several neurodegenerative diseases [1]. Numerous studies have proven the efficacy of different medicinal plants in the prevention of many neurological conditions, due to their content of antioxidant compounds [2].

Dementia, motor dysfunction, and aberrant involuntary movements are significant hallmarks of the neurological disorder Huntington’s disease (HD) [3, 4]. This disease is characterized mainly by aberrant protein accumulation and aggregation leading to disturbances in the dynamics of protein networking [5]. This occurs due to pathologic enlargement of the polyglutamine (polyQ) sequence in the N-terminal region of the huntingtin (Htn) protein forming 35 or more CAG repeats [6, 7]. Treatments available for HD currently treat only symptoms without affecting disease progression. Therefore, novel safe treatments that can act on several pathways and targets related to this disease are interesting to explore [8].

Chia, *Salvia hispanica* L., is a member of the genus *Salvia*, family Lamiaceae. It is an annual herbaceous plant native to northern Guatemala and southern Mexico, which are considered the largest producers of chia seed worldwide. Currently, it is also cultivated in other countries, such as Columbia, Australia, Peru, Bolivia, America, Argentina and Europe [9, 10]. Historically, Aztecs and Mayas were two ancient Mesoamerican societies that employed *Salvia hispanica* L. in addition to corn, beans, and amaranth for traditional medicines and food. It is extensively used in baked products such as cookies and bread, snacks, cereal bars, and salads because of its safety [11].

Chia means greasy or oily, it is called after the Spanish word (chian). It is mainly composed of proteins, fats, carbohydrates, minerals and vitamins [12–14]. Seeds also contain significant amounts of fiber so they can absorb water up to 15 times their weight. This high fiber content enhances the peristaltic movement of the bowel, lowers the plasma cholesterol levels, and slows the breakdown and release of glucose, all of which are beneficial in the management of metabolic diseases [11].

Moreover, chia oil consists of ω-6 and ω-3 fatty acids at a ratio of 0.3:0.35. This ratio is very important for decreasing cholesterol levels, preventing inflammation and enhancing cognitive ability [10, 15]. This seed is rich in polyphenolic compounds with antioxidant activities, such as kaempferol, rosmarinic acid and caffeic acid [16]. These antioxidants have previously shown protective properties against the deleterious action of reactive oxygen and nitrogen species, which are involved in aging and many metabolic and neurodegenerative disorders [17].

Chia is also commonly used as a dietary supplement because of having plethora of benefits, such as strengthening bones and muscles, supporting the digestive system and lowering the risk of diabetes and heart diseases [11, 18, 19]. In terms of neuroprotective potential, chia seed oil has neuroprotective effects through decreasing inflammation and oxidative stress in the rat brain [20]. Moreover, the oil in the form of nanoemulsion could protect against oxidative stress and motor impairment induced by rotenone in a Parkinson’s disease mouse model [21]. To the best of our knowledge, the neuroprotective properties of chia seed against HD development have not been evaluated yet.

The use of *Caenorhabditis elegans* (*C. elegans*) worm, a straightforward model that is easy to manipulate, can be very advantageous for studying the antioxidant and neuroprotective properties of chia seed. Their short lifespan and life cycle allow their use in assessing neurodegenerative diseases, such as HD, in a quick manner [22]. Furthermore, many studies have demonstrated a high degree of molecular and cellular pathway conservation between mammals and worms [23]. In fact, a comparison of the genomes of humans and *C. elegans* revealed that most human illness genes and disease pathways are also present in *C. elegans* [24].

It is also a useful model for comprehending the molecular processes regulating stress responses and aging. To better understand protein misfolding and aggregation, a number of aggregation-prone human proteins linked to neurodegenerative disorders have been produced in a range of *C. elegans* tissues [25–27].

In our current study, the *C. elegans* model was used to test the antioxidant and neuroprotective effects of chia seed dichloromethane (DCM) fraction on the development of HD. The metabolome characterization was done using a chromatographic hyphenated ultra-performance liquid chromatography coupled to mass spectrometry UPLC‒q-TOF‒ESI‒MS/MS approach. The oxidative stress resistance and antioxidant abilities of the fraction were tested via survival and ROS assays. The involvement of DAF-16 pathway, with its downstream target genes was tested by conducting DAF-16 localization, SOD-3 and HSP-16.2 expression assays. The neuroprotective effect against HD-associated phenotypes was evaluated through studying the ability of the extract to reduce the formation of polyQ150, polyQ35 and polyQ40 aggregates.

## Materials and methods

### Plant material, extraction and fractionation

Seeds of *Salvia hispanica* L. were purchased from Harraz Herbal drug store, Cairo, Egypt and authenticated by Agr. Eng. Treasa Labib at the Center for Documentation of Cultural and Natural Heritage, Egypt. A voucher specimen (Sl2021) was deposited in the Pharmaceutical Biology Herbaruim, Faculty of Pharmacy and Biotechnology, German University in Cairo, Egypt.

Two kilograms of *Salvia hispanica* seeds were ground and extracted with a homogenizer-assisted technique at a 1:4 methanol/water ratio for 3 h at room temperature and 600 rpm. Methanol was chosen as the extraction solvent to ensure high efficiency and maximum yield. The filtered extract was subjected to vacuum evaporation via a rotary evaporator (BUCHI, R-210; Switzerland) at 60 °C to yield a final dry extract of 37.5 w/w%.

Solvent–solvent fractionation was carried out in order of increasing polarity, yielding 4.1 w/w% of the DCM fraction. The concentrated DCM fraction was then lyophilized and stored at -20 °C until further use. The fraction was dissolved in 70% ethanol for use in worms, and new solutions were made and utilized each time.

### Chemicals, reagents and *C. elegans* strains

The *C. elegans* strains used in this study and *Escherichia coli* (*E. coli* OP50) were obtained from IPMB, Heidelberg, Germany. The strains used were as follows: wild-type N2, CF1038 (*daf-16(mu86)I*), TJ356 (*zIs356 [daf-16p::daf-16a/b::GFP + rol-6]*), TJ375 (*gpIs1[hsp-16.2::GFP]*), CF1553 (*mu1s84[pAD76(sod-3::GFP)]*), AM140 (*rmIs132[unc-54p::Q35::YFP]*), AM141 (*rmls133[unc-54p::Q40::YFP]*) and HA759 (*rtIs11[osm-10p::GFP+osm10p::HtnQ150 + Dpy-20(+)]*). Worms were cultured in Nematode Growth Medium (NGM) plates with *E. coli* OP50 as a food source in 20°C incubator. L1 synchronized worms were obtained from a uniformly aged population through bleaching of gravid adults with 5% NaOCl and 5 M NaOH (3:1) [28]. The reagents used in the current study were purchased from Sigma‒Aldrich, Germany. They are: 2’,7’-dichlorodihydrofluorescein diacetate (H_2_DCF-DA), benzaldehyde (98%), and 5′-fluorodeoxyuridine (FUDR, 98%), which is utilized to block progeny production; juglone, which is used for inducing oxidative stress; and sodium azide, which is used for paralysis induction. S-medium, in which the worms were routinely maintained, was prepared as follows: S Basal (5.85 g NaCl, 1 g K_2_HPO_4_, 6 g KH_2_PO_4_, 1 ml cholesterol solution [5 mg/ml in ethanol], and distilled water up to 1 L) supplemented with 10 ml of 1 M potassium citrate (pH 6.0), 10 ml trace metals solution, 3 ml of 1 M CaCl_2_, and 3 ml of 1 M MgSO_4_.

### Assessment of oxidative stress resistance

This experiment was performed, as previously described, using worms of the wild-type (N2) and CF1038 (*daf-16* null mutants). It tests the ability of CH seed to protect from oxidative stress [29]. CF1038 strain was used to assess whether the absence of the DAF-16/FOXO (human fork head transcription factor) pathway will affect the activity of the tested fraction or not. Age-synchronized L1 worms were divided into six groups of approximately two hundred worms each. Three groups served as negative controls, two groups did not receive any treatment (untreated control groups), and one group was the solvent control group treated with 0.32% ethanol, the solvent used to dissolve the CH seed DCM fraction. The other three groups were given CH seed DCM fraction at various concentrations of 50, 100 and 200 µg/ml. All groups were kept in S-medium and *E. coli* OP50 (OD_600_ = 1.0) at 20 °C. Following the 48 h treatment period, the pro-oxidant juglone was introduced at a lethal final concentration of 80 µM to all groups except one of the untreated control groups. This was followed by a 24 h incubation period at 20 °C. The number of living and dead worms was then determined via a stereozoom microscope (Optika SLX3, Italy). Worms that did not move upon stimulation with a platinum wire were considered dead. The experiment was performed three times, and the data were presented as the mean of survival percentage ± SEM. The results were compared and presented via GraphPad Prism (5.01), and significance was measured via one-way ANOVA followed by Bonferroni’s method (post hoc).

### Intracellular ROS measurement

For further evaluation of the antioxidant capability of CH seed, the intracellular endogenous ROS levels were measured. L1, N2, worms were used for this experiment. They were sorted into groups of five and treated with CH seed DCM fraction at concentrations of 50, 100 and 200 µg/ml. Two groups were used as negative control groups: an untreated control group and a solvent control group (0.32% ethanol). S-medium + *E. coli* OP50 was added to all groups of worms starting the L1 stage, and they were kept at 20 °C for 72 h. Afterwards, M9 buffer was used for washing, and H_2_DCF-DA dye was added at a concentration of 50 µM to all groups. All worms were incubated for 1 h in darkness at 20 °C. Before reading, the worms were rinsed with M9 buffer for removal of any extra dye. Reading was performed using microscopic glass slides and 10 mM sodium azide to induce paralysis. An Axiostar Plus 37081, Carl Zeiss, fluorescence microscope was used to view approximately twenty worms/group at 10X magnification and constant exposure time. The results of three independent assays are expressed as the mean fluorescence intensity and were compared via one-way ANOVA followed by Bonferroni’s (post-hoc) method. The mean fluorescence intensity for the whole body was measured using ImageJ software (Version 1.48, National Institutes of Health, Bethesda, MD, USA). The assay was repeated three times and the results were analyzed and compared with one-way ANOVA followed by Bonferroni’s (post-hoc) method [29].

### DAF-16::GFP localization

TJ356 transgenic worms were used in this experiment. These nematodes express DAF-16 coupled with the green fluorescent protein (GFP). One day after bleaching, L1 worms were divided into five groups and treated as described above in the ROS assay. All the groups were left for 24 h in S-medium and *E. coli* OP50 at 20 °C, after which the DAF-16::GFP distribution inside the bodies of the worms was recorded [28]. Twenty to thirty worms were mounted on glass slides, paralyzed with sodium azide, and visualized at constant exposure times and 20X magnification via a fluorescence microscope.

The experiment was performed four independent times, and the nematodes were sorted according to their DAF-16::GFP localization to cytosolic, nuclear or intermediate. The percentage of worms with nuclear localization was calculated. Data comparison was performed as described above.

### SOD-3 expression

Age-synchronized, L1, CF1553 transgenic nematodes were used in this experiment. These worms expressed superoxide dismutase 3 fused to GFP (SOD-3::GFP). The five groups were treated as described above, and they were incubated with *E. coli* OP50 and S-medium at 20 °C. After three days, a fluorescence microscope was used to capture approximately 30 photos of the worms via a 10X objective lens at a constant exposure time. The mean fluorescence intensity of the posterior intestine was measured over the course of three different experiments via ImageJ software. Results are presented as the mean fluorescence intensity [30]. A comparison of the results was performed as described for the ROS assay.

### Hsp-16.2 expression

L1, TJ375, worms were used in this assay. These worms carry HSP-16.2::GFP. Worms were grouped into groups of five, and treatment was performed as described for the ROS assay. All the groups were kept at 20 °C for 72 h. Afterwards, a subtoxic dose of juglone (20 µM) was added to all the groups. The samples were left for 24 h at 20 °C, after which reading was performed. Approximately 20 worms/group were photographed at 20X magnification with a constant exposure time after being paralyzed by sodium azide. The assay was performed three times, and ImageJ was used to measure the fluorescence intensity of the head and pharynx. Data analysis and comparison were performed as described above.

### Neuronal survival

Neuronal survival was assessed via the HA759 strain. Throughout aging, these worms express Htn-Q150 linked to GFP in ASH sensory neurons located in their heads. Over time, these neurons gradually lose their function due to the formation of Htn-Q150 aggregates. Worms expressing only one fluorescent neuron, instead of two, are believed to be suffering from neurodegeneration [31]. After synchronization, L1 worms were divided into five groups, as described above, and then left in S-medium and *E. coli* OP50 for three days at 20 °C. Worms were collected, placed on a glass slide, and rendered immobile using sodium azide on the day of reading. A fluorescence microscope was used for scoring the worms with live and dead ASH neurons (those with 2 fluorescent neurons and 1 fluorescent neuron, respectively) using a consistent exposure time and a 10X objective lens. Approximately 30 nematodes were observed per group. The data are presented as the means of three distinct trials. The percentage of neuronal viability was calculated as the percentage of surviving ASH neurons. A comparison of the results was performed as mentioned above.

### Chemosensory assay

The purpose of this assay was to evaluate the chemosensory response of HA759 worms to assess the survival of ASH neurons, as mentioned previously [32]. Five groups, approximately 100 L1 nematodes each, were divided and treated as described above. Following treatment, the worms spent three days in S-medium and *E. coli* OP50 in a 20 °C incubator. NGM chemotaxis plates (60 mm without food), which were divided into two exact equal parts (A and B), were used in this assay. To cause paralysis of the worms on each side, a drop of 0.5 M sodium azide was added to the edge of both sides. Part A received a drop of 0.1% benzaldehyde in 99.8% ethanol at the edge as an attractant to the worms. Part B received a drop of 99.8% ethanol as a control. To remove any remnants of *E. coli* OP50, the worms were rinsed three times with M9 solution just before the experiment was conducted. Afterwards, the worms were placed in a spot in the center of each plate and kept in an incubator at 20 °C. Following the 1 h period, the number of worms in every quadrant was counted via a stereozoom microscope, as mentioned above, and the chemosensory index was calculated using the following formula:

Chemosensory index = number of worms in A - number of worms in B/collective number in A + B.

The experiment was conducted three times, and the results are expressed as the means ± SEMs. Data comparison was performed as described above.

### PolyQ35 cluster formation

In this study, the AM140 strain with polyQ35 linked to yellow fluorescent protein (polyQ35::YFP) was used. The purpose of this experiment was to explore polyQ35 aggregation, which is age dependent and typically begins on the third day of adulthood. This happens in worms when the protein begins to change from a soluble to a clustered state that builds up in the body wall muscles [33]. As previously mentioned, worms were split into five treatment groups at the L1 stage and added to S-medium and *E. coli* OP50 in a 20 °C incubator. FUDR (160 µM) was used to halt progeny in worms at the L4 stage. Worms were evaluated for the number of polyQ35 clusters after five days of treatment. The samples were collected on the day of reading and subjected to the previously mentioned fluorescence microscopy analysis using a constant exposure time and 10X magnification. The number of polyQ35 clusters was approximately 30 worms/group. The experiment was performed three times, and the results are presented as the mean number of clusters ± SEM. The analysis was performed as described above.

### PolyQ40 aggregation

Age-synchronized AM141 transgenic worms (polyQ40::YFP) were used in this study to test the formation of polyQ40 aggregates. Worms were treated during the L1 stage after being divided into five experimental groups, as described above. They were set up and kept in S-medium + *E. coli* OP50 at 20 °C for five days. FUDR was used as described for the polyQ35 cluster assay to halt progeny. Fluorescence microscopy was used to visualize the worms via a 10X lens with a constant exposure time. Images of approximately thirty worms were captured. The number of polyQ40::YFP aggregates present within the muscles was counted throughout the worms’ bodies during three separate rounds of testing. The mean number of aggregates ± SEM is presented and the results were compared in the manner mentioned above.

### Body bending frequency assay

Both the AM140 and AM141 strains were used in this assay to measure body bending, which is an indicator of the locomotive properties of the nematodes. L1 worms were divided into five groups as described in previous assays and left in S-medium + *E. coli* OP50 in a 20 °C incubator. The addition of FUDR was performed when the worms reached the L4 stage to avoid having progeny. Measurements were performed on days 1, 5 and 10 of adulthood. First, the worms were washed with M9 buffer and allowed to recover for 120 s. Second, they were added to NGM plates devoid of food. Body bends were counted over duration of 30 s via a stereozoom microscope, as mentioned in oxidative stress resistant assay. The body bend is counted on the basis of the movement of the body of the worm in a sinusoidal way. Approximately 50 worms from each group were observed. The assay was performed three times and the results are shown as the mean number of bends/30 s ± S.E.M.

### Chemical profiling of the constituents via UPLC‒q-TOF‒ESI‒MS/MS

An Evosphere C18 column with a particle size of 1.7 μm (100 × 2.1 mm) was used in this study. The mobile phase included two solvents, (A) water containing 0.1% formic acid and (B) methanol containing 0.1% formic acid, with a total flow rate of 0.3 ml/min. A 53-min gradient was used for chromatographic separation. Initially, the column was equilibrated with 10% B for 3 min. The percentage of B was subsequently increased to 60% within 27 min and maintained for another 2 min, followed by a further increase to 90% over 2 min, which was maintained isocratically for an additional 3 min. The conditions were then returned to 10% B for 2 min, and the column was re-equilibrated at 10% B for an additional 3 min before the next injection.

For mass spectral analysis, a q-TOF high-resolution mass spectrometer with an electrospray ionization (ESI) interface in negative ionization mode was employed. The mass spectrometer characteristics were as follows: interface voltage of 4.0 kV, interface temperature of 300 °C, nebulization and drying gas flow rate of 3 L/min, heat block temperature of 400 °C, DL temperature of 280 °C, detector voltage of 1.8 kV, and flight tube temperature of 42 °C.

To achieve high mass accuracy, sodium iodide was utilized as a calibration solution. MS¹ and MS² spectra were collected simultaneously via data-dependent acquisition (DDA) for all ions with *m/z* values ranging from 100 to 1000 Da and an intensity threshold over 5000. MS² tests utilized argon as the collision gas, with collision energy of 30 eV and a spread of 5 eV. NaI clusters were utilized for equipment calibration, ensuring a mass accuracy of 1 ppm throughout the investigation [34].

## Results

### Oxidative stress resistance activity of CH seed

**Fig. 1:**
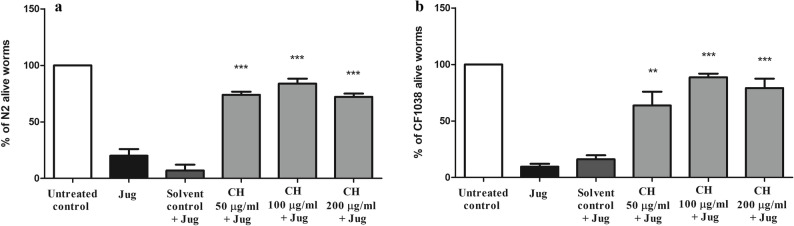
CH seed increases tolerance to oxidative stress mediated by juglone in N2 and CF1038 worms. **a** In N2 worms, compared with the solvent control + juglone group, the CH 50 µg/ml, CH 100 µg/ml and CH 200 µg/ml groups presented significantly greater survival rates (***p < 0.001). **b** In CF1038 worms, compared with the solvent control + juglone group, the CH 50 µg/ml (**p < 0.05), CH 100 µg/ml and CH 200 µg/ml groups presented significantly greater survival rates (***p < 0.001). The data represent the mean survival percentage ± SEM of three different trials, 200 worms/group. Comparisons were made through one-way ANOVA followed by the post-hoc Bonferroni technique

In this assay, a deadly dose of the naphthoquinone juglone was utilized as a pro-oxidant to induce a state of severe oxidative stress in both N2 and CF1038 strains. CF1038 strain is a *daf-16* null mutant. It lacks DAF-16 transcription factor that regulates many genes involved with longevity and stress response [35]. For N2 strain, compared with those in the solvent control + juglone group, the survival rates of the worms treated with CH seed increased dramatically. Compared with the solvent control + juglone (12.07 ± 4.1%), the CH 50 µg/ml, CH 100 µg/ml and CH 200 µg/ml groups presented highly significant survival rates of 73.98 ± 2.8%, 83.87 ± 4.3% and 72.15 ± 2.8%, respectively (***p < 0.001) (Fig. 1a). For CF1038 strain, in comparison with the solvent control + juglone group (16.11 ± 3.6%), all CH seed treated worms showed significant survival increase. The CH 50 µg/ml, CH 100 µg/ml and CH 200 µg/ml groups presented highly significant survival rates of 63.72 ± 12.3% (**p < 0.05), 88.65 ± 3.3% and 79.13 ± 8.3%, respectively (***p < 0.001) (Fig. 1b**)**. This assay also confirmed the bioavailability of the DCM fraction inside the worms and demonstrated its safety.

### CH seed decreases intracellular ROS levels

**Fig. 2:**
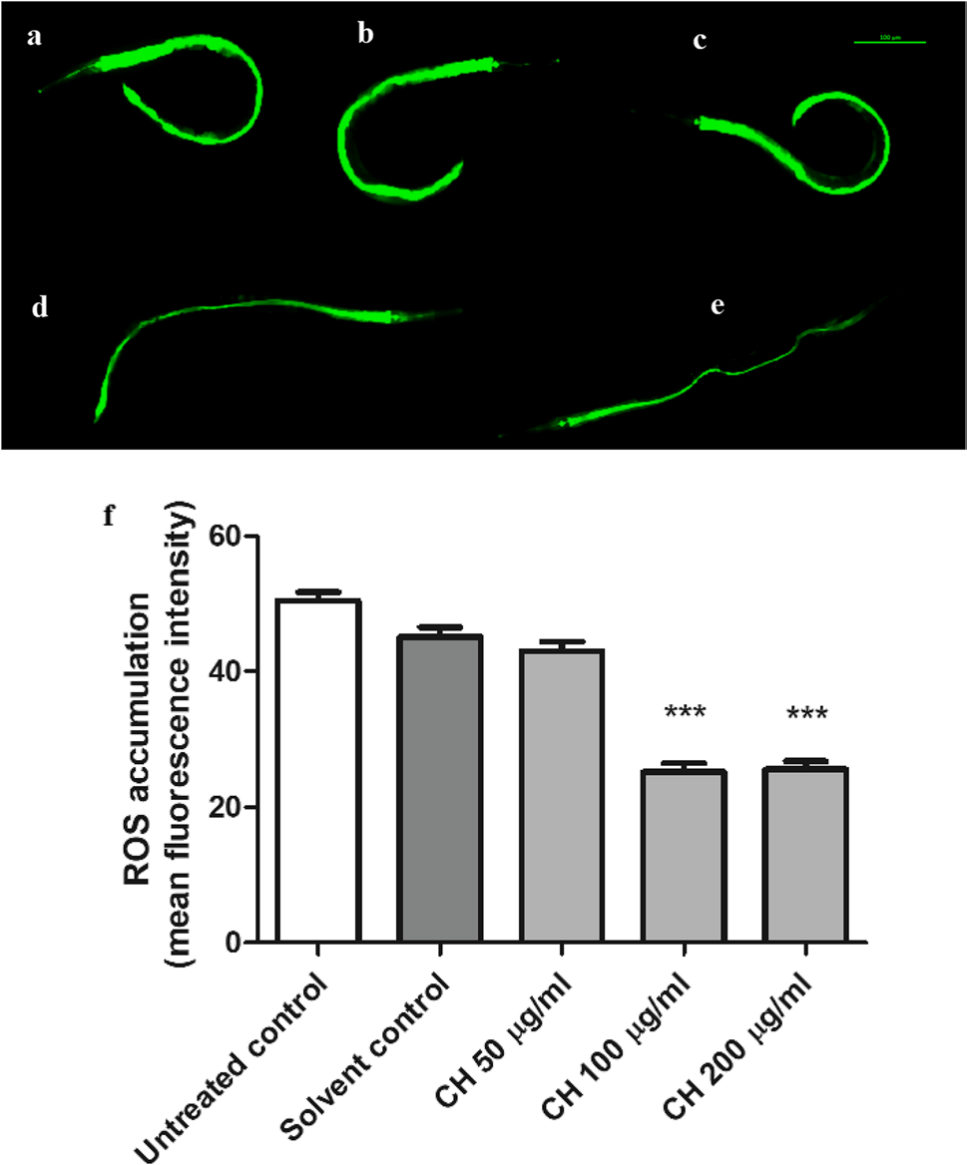
CH seed decreases ROS levels in N2 worms. **a** Untreated control. **b** Solvent control. **c** CH 50 µg/ml. **d** CH 100 µg/ml. **e** CH 200 µg/ml. **f** The CH 100 µg/ml and CH 200 µg/ml groups presented significantly lower ROS levels than did the solvent control group (***p < 0.001). Scale bar, 100 μm. The data represent the mean fluorescence intensity ± SEM of three different trials, 20 worms/group. Comparisons were made through one-way ANOVA followed by the post-hoc Bonferroni technique

This assay was performed to detect the effect of the CH seed on the basic intracellular ROS levels inside wild-type N2 worms. H_2_DCFDA, a membrane permeable dye, was used in this assay for detection. When the dye enters the cell, the intracellular esterases deacetylate it. The oxidation of intracellular ROS results in the formation of the 2′,7′-dichlorofluorescin state, which is highly fluorescent. The fluorescence intensity reflects the levels of ROS. CH seed was able to decrease the basal ROS levels inside the worms, as detected by a reduction in fluorescence intensity. Compared with the solvent control, the 100 µg/ml CH seed treatment resulted in a 44.20 ± 1.3% decrease in fluorescence intensity. Compared with the solvent control, 200 µg/ml CH seed treatment led to a 43.36 ± 1.2% reduction in fluorescence intensity (***p < 0.001) (Fig. 2).

### CH seed increases DAF-16 nuclear localization

**Fig. 3:**
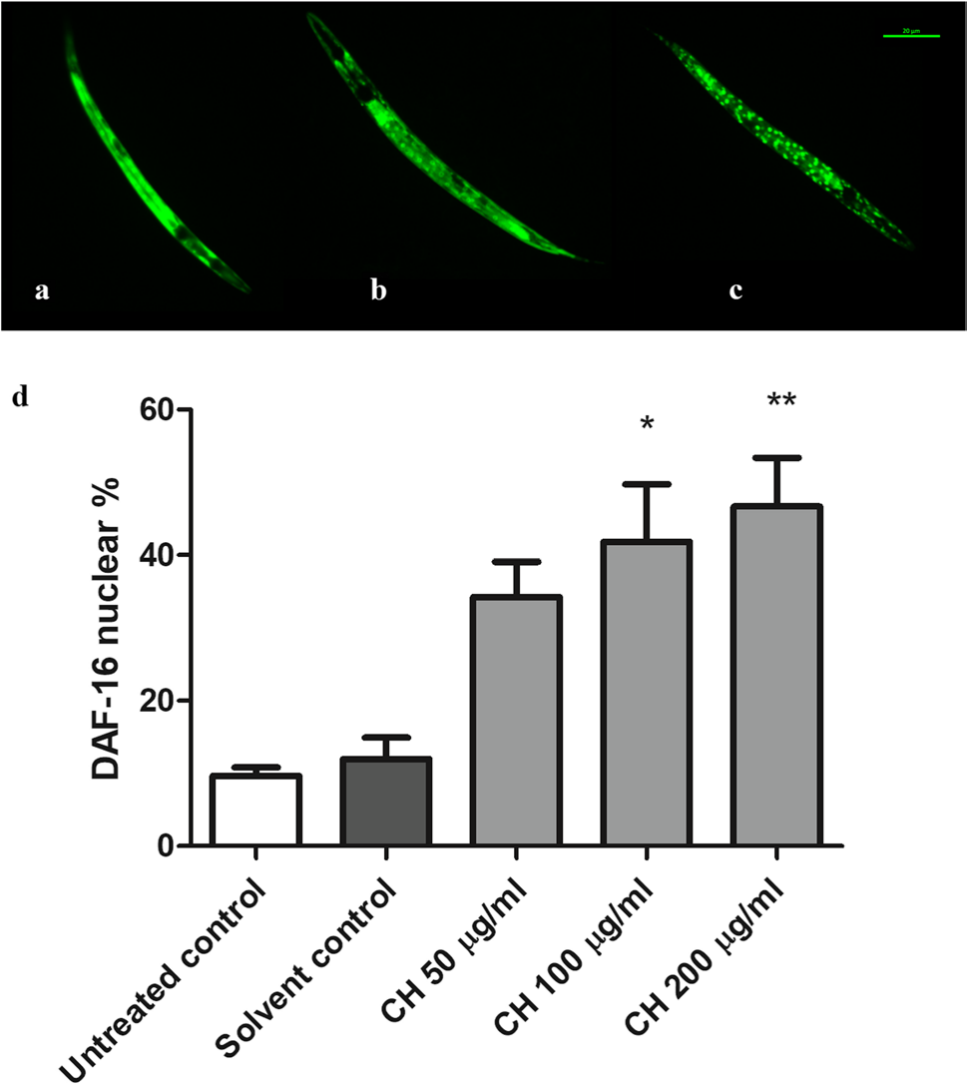
CH seed promotes DAF-16 nuclear localization. **a** Cytoplasmic localization. **b** Intermediate localization. **c** Nuclear localization. Scale bar, 20 μm. **d** Data showing the percentage of worms with nuclear localization. CH (100 µg/ml) and CH (200 µg/ml) increased the translocation of DAF-16::GFP in TJ356 mutant worms to the nucleus. The data are shown as the means ± SEMs for four different experiments, 20–30 worms/group, and were analyzed via one-way ANOVA followed by the Bonferroni correction (post-hoc). *p < 0.05 and **p < 0.01 compared with the solvent control group

DAF-16 experiment was done to determine the contribution of this pathway in the oxidative stress resistance activity of CH seed. The FOXO and the *C. elegans* DAF-16 transcription factor are homologs. Under normal conditions, DAF-16 is found in the cytosol in its phosphorylated inactive state. Stress and other environmental conditions trigger its dephosphorylation leading to its nuclear localization. Once activated, it regulates many genes involved in the stress response and lifespan extension [36]. In this assay, worms treated with CH seed demonstrated noticeably greater nuclear localization of DAF-16 than did those in the solvent control + juglone group. CH (100 µg/ml) and CH (200 µg/ml) resulted in nuclear localizations of 41.81 ± 7.8% (p* < 0.05) and 46.66 ± 6.7% (p** < 0.01), respectively, while the percentage in the solvent control group was 11.98 ± 2.9% (Fig. 3). This result strongly suggests the involvement of the DAF-16/FOXO pathway in the in vivo oxidative stress resistance activity of CH seed.

### CH seed increases SOD-3 expression

SOD-3 is an additional stress response gene that is directly activated as a result of DAF-16 activation. It scavenges the superoxide anion radical, O_2_^−^, to protect against high ROS levels [37]. An increase in its level has been previously reported with many antioxidants [28]. Compared with that of the solvent control group, the fluorescence intensity of the worms treated with CH seed was significantly greater.

**Fig. 4:**
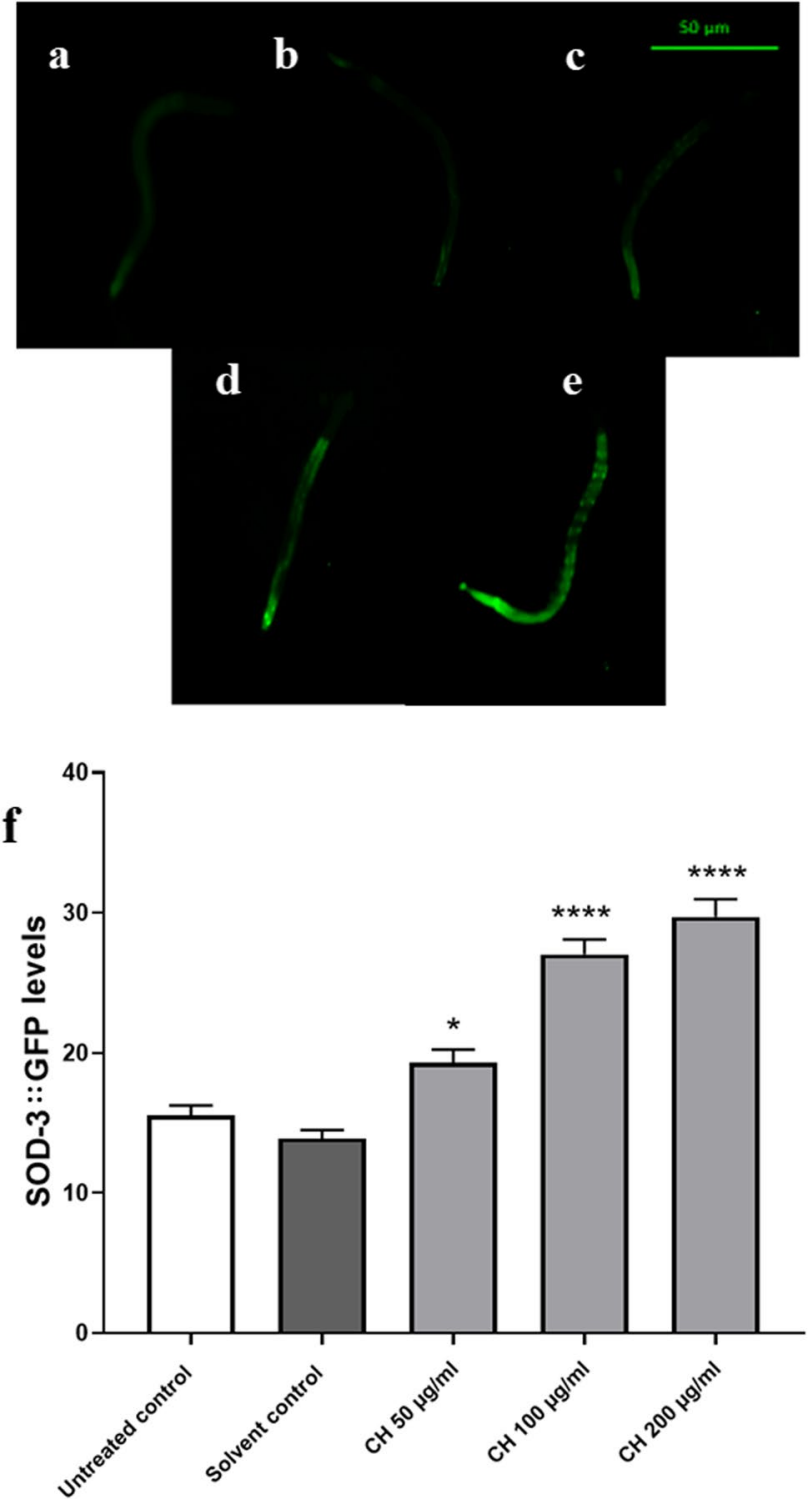
CH seed increases SOD-3 levels in CF1553 worms. **a** Untreated control. **b** Solvent control. **c** CH 50 µg/ml. **d** CH 100 µg/ml. **e** CH 200 µg/ml. **f** The CH 50 µg/ml, CH 100 µg/ml and CH 200 µg/ml groups presented significantly greater SOD-3 levels than did the solvent control group (*p < 0.05, ****p < 0.0001). Scale bar, 50 μm. The data represent the mean fluorescence intensity ± SEM of three different trials, 30 worms/group. Comparisons were made through one-way ANOVA followed by the post-hoc Bonferroni technique

50 µg/ml CH increased the fluorescence intensity by 26.88 ± 0.5%, 100 µg/ml CH and 200 µg/ml CH increased the fluorescence intensity by 103.88 ± 1.0% and 126.35 ± 1.2%, respectively (*p < 0.05 and ****p < 0.0001) (Fig. 4).

### CH seed decreases HSP expression after juglone exposure

**Fig. 5:**
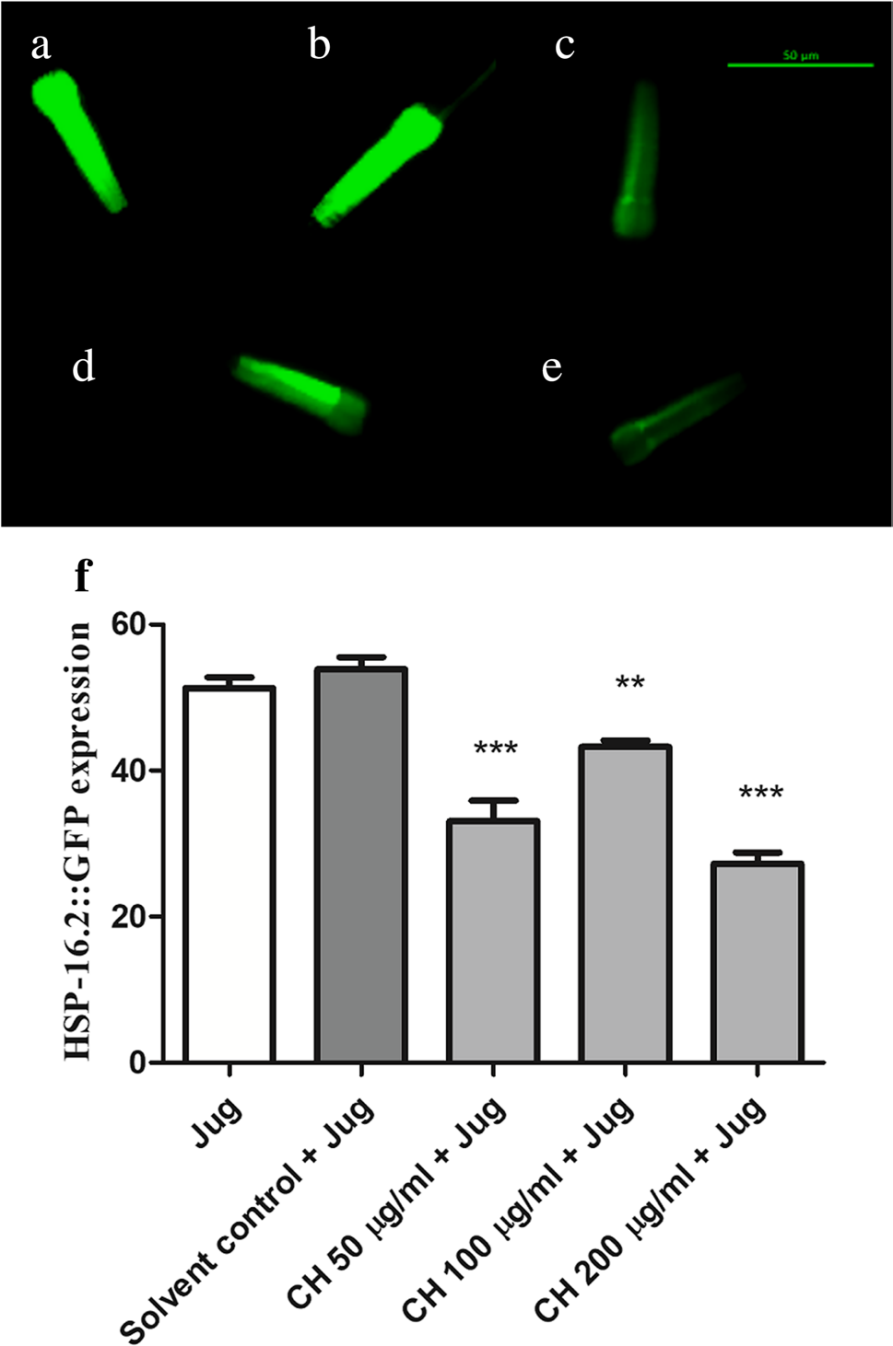
CH seed decreases HSP-16.2 levels in TJ375 worms. **a** Juglone. **b** Solvent control + Juglone. **c** CH 50 µg/ml + Juglone. **d** CH 100 µg/ml + Juglone. **e** CH 200 µg/ml + Juglone. **f** The CH 200 µg/ml + Juglone group presented significantly lower ROS levels than did the solvent control + Juglone group (***p < 0.001). Scale bar, 50 μm. The data represent the mean head and pharynx fluorescence intensity ± SEM of three different trials, 20 worms/group. Comparisons were made through one-way ANOVA followed by the post-hoc Bonferroni technique

To further assess the antioxidant capacity of CH seed, HSP was quantified in TJ375 worms expressing HSP-16.2::GFP in their head and pharynx. Its expression is inducible by both heat and oxidative stress. Worms were first treated, as mentioned above, and then given 20 µM juglone to induce a mild state of oxidative stress. Compared with the solvent control + jug group, the CH 50 µg/ml + jug group presented a decrease in fluorescence intensity of 38.64 ± 2.7%, the CH 100 µg/ml + jug group presented a decrease of 19.75 ± 0.8%, and the CH 200 µg/ml + jug group presented the greatest decrease in fluorescence intensity (49.53 ± 1.5%) (**p < 0.01 and ***p < 0.001) (Fig. 5).

### CH seed exerts neuroprotective effects on ASH neurons

**Fig. 6:**
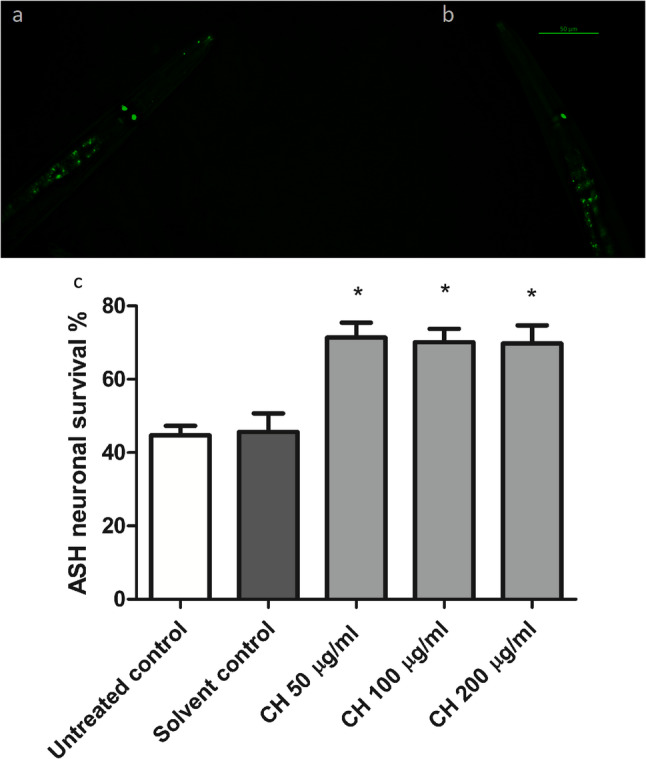
Effects of CH seed on ASH neuronal survival in HA759 worms. **a** Photo of HA759 worm with bilateral GFP fluorescent neurons. **b** Worm with only one surviving ASH neuron. Scale bar, 50 μm. **c** Survival rate of ASH neurons after CH seed treatment. The data are presented as the means ± SEMs of three independent experiments, 30 worms/group, and were analyzed via one-way ANOVA followed by the Bonferroni correction (post-hoc). *p < 0.05 compared with the solvent control group

The *C. elegans* polyQ150 strain HA759 was utilized to test the ability of CH seed to protect ASH neurons from polyQ150-mediated death. GFP fluorescence in both types of ASH neurons is used as an indicator of their survival. After three days of treatment, ASH neurons presented a high mortality rate in the solvent control group, with one surviving neuron rather than two surviving neurons, in contrast to the CH seed treated groups. In the solvent control group, only 45.66 ± 5.0% ASH neuronal survival was observed, whereas it increased to 71.35 ± 4.0%, 70.12 ± 3.6% and 69.77 ± 4.9% in the groups treated with 50 µg/ml CH, 100 µg/ml CH and 200 µg/ml CH, respectively (*p < 0.05) (Fig. 6).

**Fig. 7:**
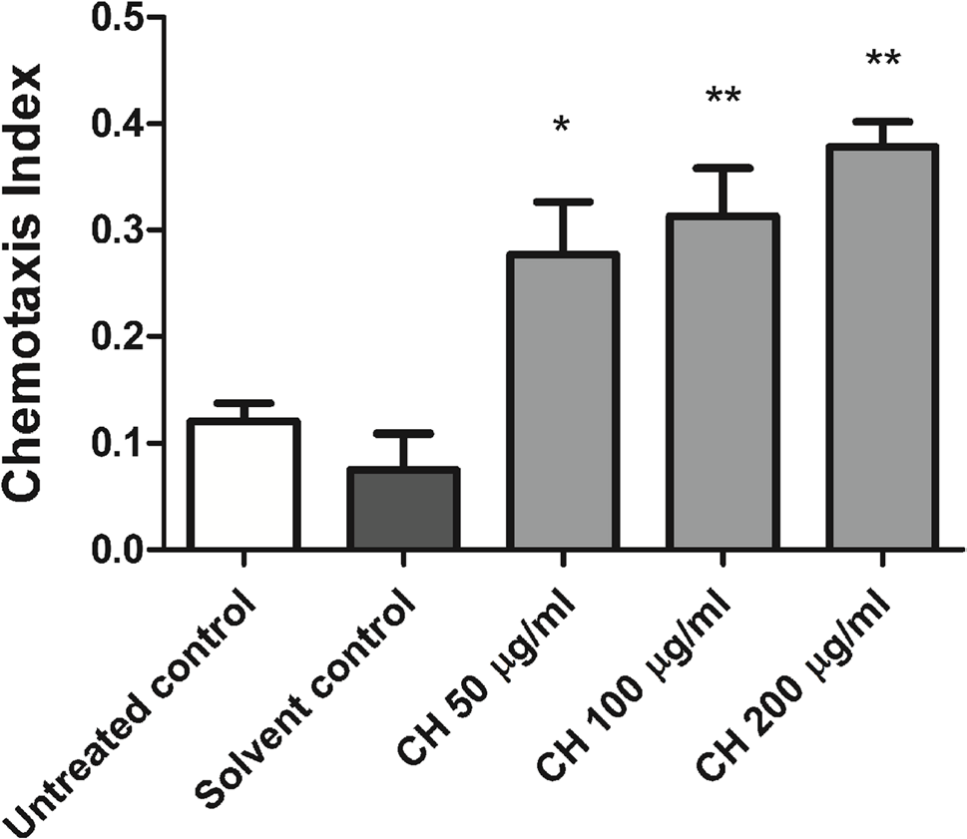
Effects of CH seed on ASH neurons via a chemotaxis assay using HA759 worms. Compared with the solvent control, CH seed significantly increased the chemotaxis index. The results are presented as the means ± SEMs of three experiments, 100 worms/group. The data were analyzed through one-way ANOVA followed by the Bonferroni correction (post-hoc). *p < 0.05 and **p < 0.01 versus the solvent control group

Since *C. elegans* chemotaxis behavior, which is mediated via ASH neurons, is influenced by odorants such as benzaldehyde, polyQ150 aggregate accumulation results in impaired ASH neuronal function and subsequently a defective chemotaxis response towards the benzaldehyde odor. A chemotaxis assay was further used to assess the potential of CH seed to protect ASH neurons. CH seed improved the behavioral deficiency induced by polyQ150 aggregates. Compared with the solvent control, treatment with 50 µg/ml CH increased the chemotaxis index by 285.7% (*p < 0.05). Additionally, the chemotaxis index was highly increased in the 100 µg/ml CH and 200 µg/ml CH treatment groups by 342.8% and 428.5%, respectively (**p < 0.01) (Fig. 7). The results collectively demonstrated that CH seed effectively reduced polyQ150 aggregation in ASH neurons in HA759 worms.

### CH seed decreases the number of polyQ35 clusters

**Fig. 8:**
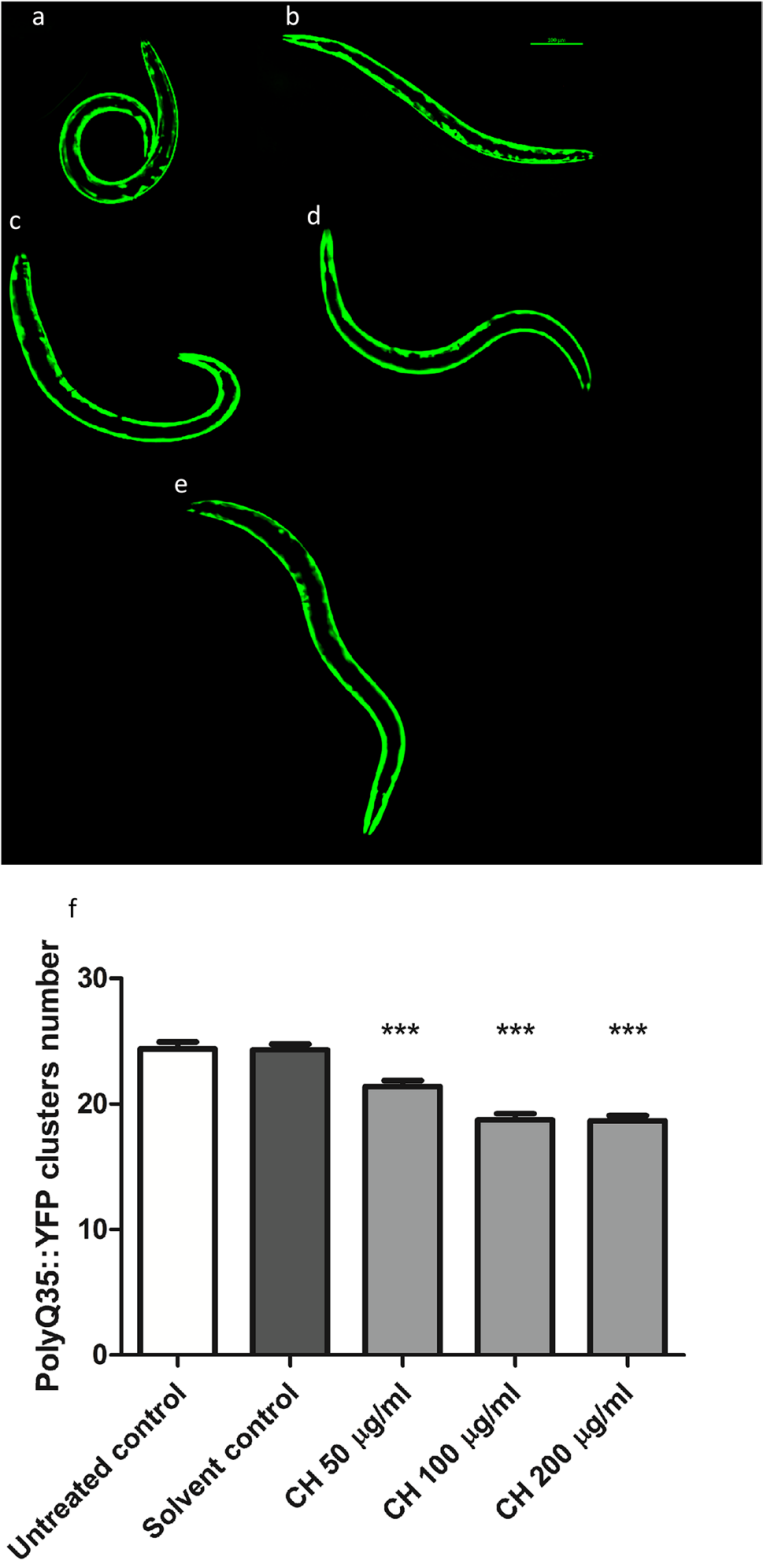
Effects of CH seed on polyQ35 cluster formation in muscle cells of the body wall in AM140 worms. **a** Untreated control. **b** Solvent control. **c** CH 50 µg/ml. **d** CH 100 µg/ml. **e** CH 200 µg/ml. Scale bar = 100 μm. **f** At all the tested doses, CH seed decreased the number of polyQ35 clusters. The results are denoted by the mean cluster number ± SEM for three different trials, 30 worms/group. Data analysis was performed with one-way ANOVA followed by the Bonferroni correction (post-hoc). ***p < 0.001 versus the solvent control group

In the AM140 strain, CH seed treated worms presented a significant decrease in the number of polyQ35 aggregates. Compared with the solvent control, the CH seed fraction at 50 µg/ml decreased aggregate formation by 12.01%, whereas the CH fraction at 100 µg/ml and 200 µg/ml resulted in a 22.9% and 23.2% decrease in the number of aggregates, respectively (***p < 0.001) (Fig. 8). This implies that a decrease in polyQ aggregation might be the reason behind the reduction in polyQ toxicity caused by the CH seed.

### CH seed reduces polyQ40 aggregation

**Fig. 9:**
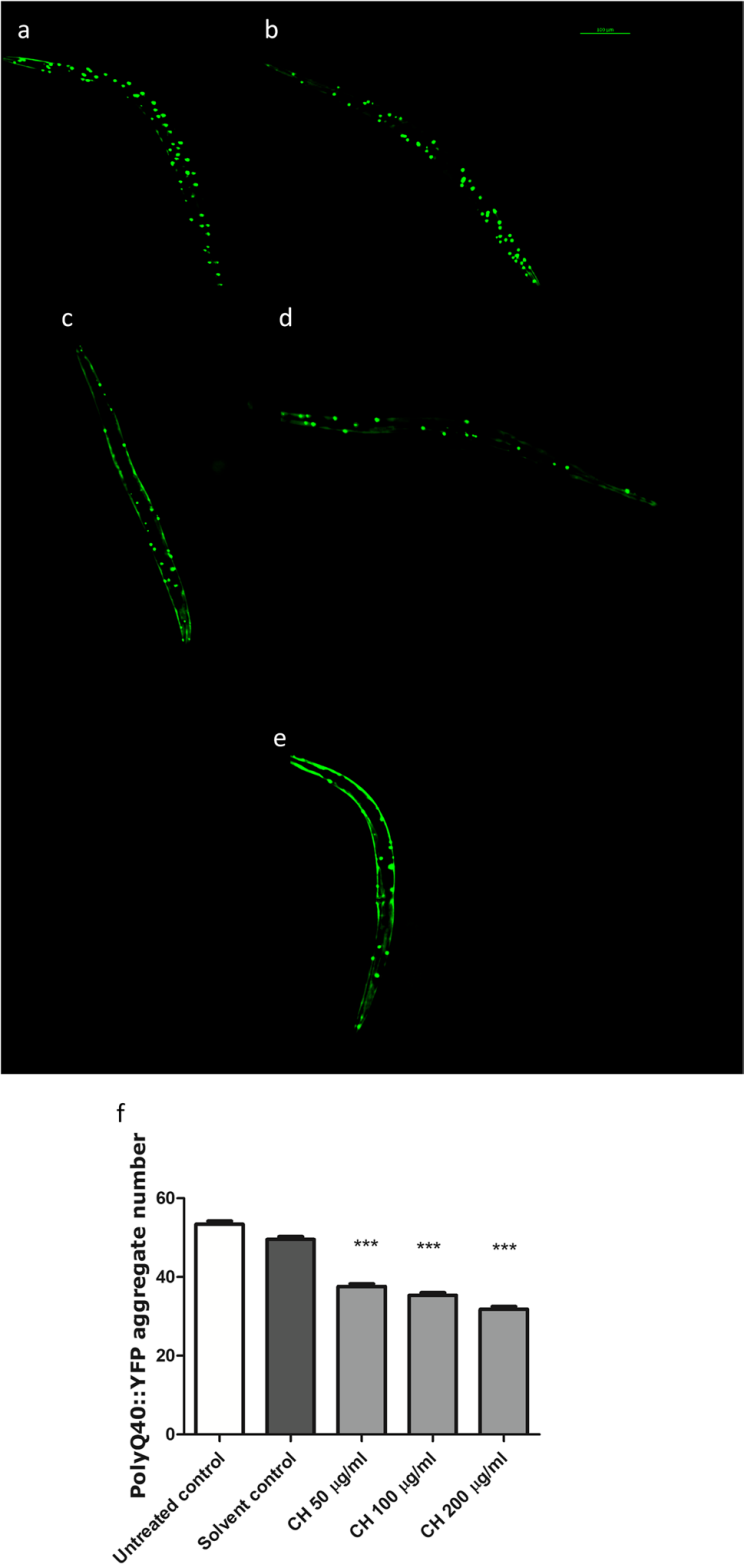
Effects of CH seed on polyQ40 aggregation in muscle cells of the body wall in AM141 worms**. a** Untreated control. **b** Solvent control. **c** CH 50 µg/ml. **d** CH 100 µg/ml. **e** CH 200 µg/ml. Scale bar = 100 μm. **f** CH seed fraction, at all the tested doses, decreased the number of polyQ40 aggregates. The results are presented as the mean aggregate number ± SEM for three independent trials, 30 worms/group. Data analysis was performed via one-way ANOVA followed by the Bonferroni correction (post-hoc). ***p < 0.001 versus the solvent control group

In the AM141 strain, the solvent control worms presented a greater number of aggregates, whereas those treated with CH seed presented a significant reduction in the number of polyQ40 aggregates. Compared with the solvent control, the CH fraction at 50 µg/ml decreased aggregate formation by 24.76%, whereas the CH fraction at 100 µg/ml and 200 µg/ml resulted in a decrease in the number of aggregates by 28.65% and 35.81%, respectively (***p < 0.001) (Fig. 9). These findings further emphasize that CH seed is able to reduce aggregation and toxicity related to polyQ aggregation.

### CH seed increases the body bending ability of AM140 worms

**Fig. 10:**
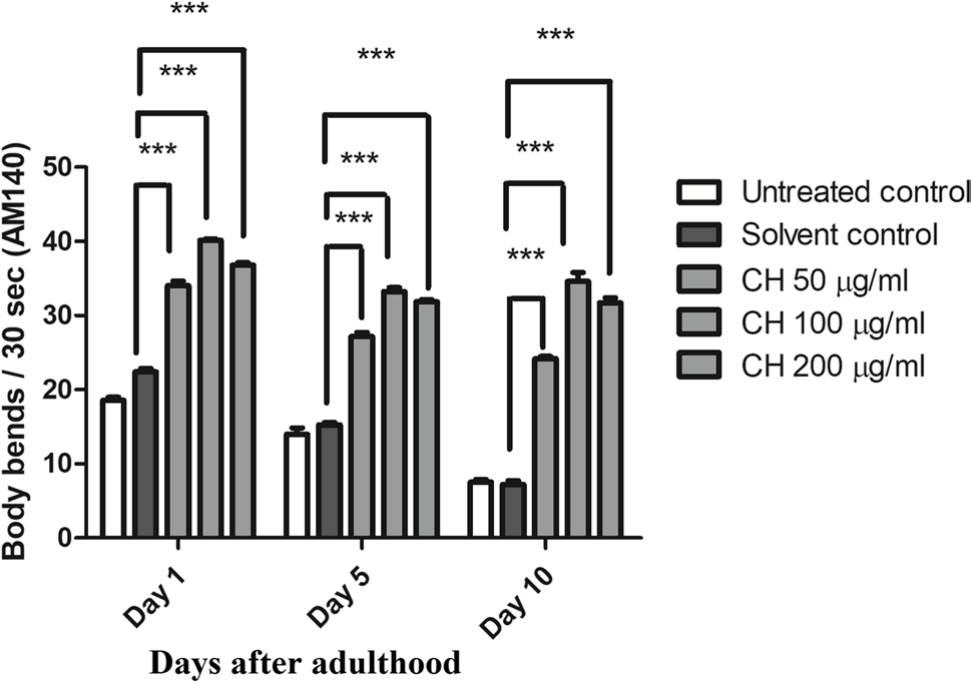
Effects of CH seed on body bending in AM140 worms. CH 50 µg/ml, CH 100 µg/ml and CH 200 µg/ml resulted in an increase in body bends at days 1, 5 and 10 of adulthood with respect to the solvent control group (***p < 0.001). The results are expressed as the mean number of body bends per 30 s ± SEM. The experiment was repeated three times with 50 worms/group and the data were analyzed via one-way ANOVA followed by the Bonferroni correction (post-hoc)

Body bending is usually used to test the locomotive ability of worms, and it decreases with age. In the AM140 strain, the number of polyQ35 clusters increased as the worms aged, leading to a decrease in overall body bends. Compared with the solvent control, CH fraction at 50 µg/ml increased the body bending ability on day 1 of adulthood by 51.83%, whereas CH at 100 µg/ml and CH at 200 µg/ml resulted in increases of 79.24% and 64.37%, respectively (***p < 0.001). Compared with the solvent control, CH seed also increased the bending ability at day 5 of adulthood in worms treated with CH at 50 µg/ml, CH at 100 µg/ml and CH at 200 µg/ml by 77.81%, 117.60% and 108.57%, respectively (***p < 0.001). Moreover, the results on day 10 of adulthood revealed an increase in the bending ability of nematodes treated with 50 µg/ml CH, 100 µg/ml CH and 200 µg/ml CH by 234.62%, 378.62% and 338.92%, respectively (***p < 0.001) (Fig. 10).

### CH seed increases the bending ability of AM141 worms

**Fig. 11:**
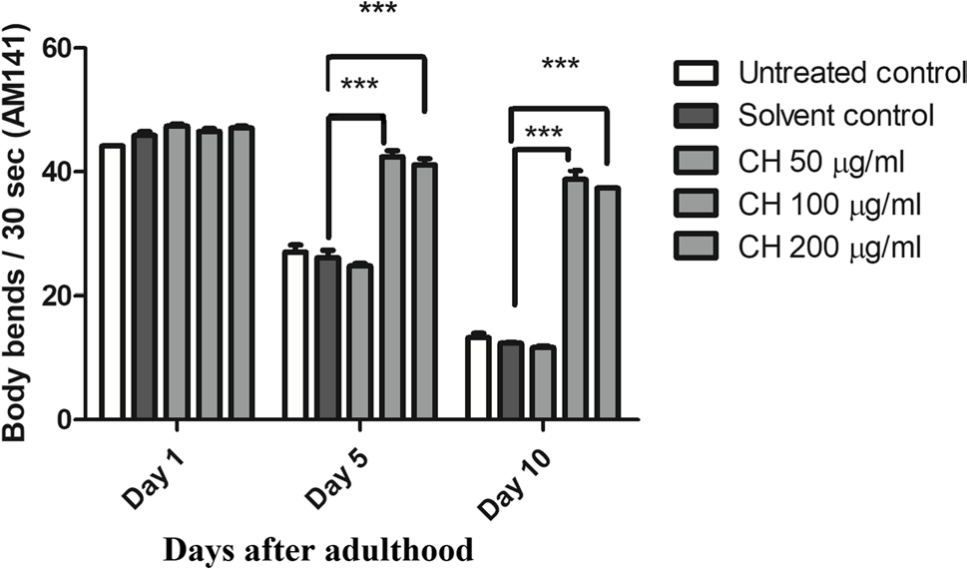
Effect of CH seed on body bending in AM141 worms. CH (100 µg/ml) and CH (200 µg/ml) resulted in an increase in body bends at days 5 and 10 of adulthood with respect to the solvent control group (***p < 0.001). The results are expressed as the mean number of body bends per 30 s ± SEM. The experiment was performed three times with 50 worms/group and the data were analyzed via one-way ANOVA followed by the Bonferroni correction (post-hoc)

In the AM141 strain, the number of polyQ40 aggregates increased with age, leading to a decrease in the thrashing frequency of the worms. Compared with the solvent control, CH seed increased the thrashing frequency on day 5 of adulthood in worms treated with 100 µg/ml CH and 200 µg/ml CH by 62.30% and 57.32%, respectively (***p < 0.001). Moreover, the results on day 10 of adulthood revealed an increase in the bending ability of nematodes treated with 100 µg/ml CH and 200 µg/ml CH by 213.41% and 202.10%, respectively (***p < 0.001) (Fig. 11).

### UPLC-q-TOF-ESI-MS/MS metabolite profiling

**Fig. 12:**
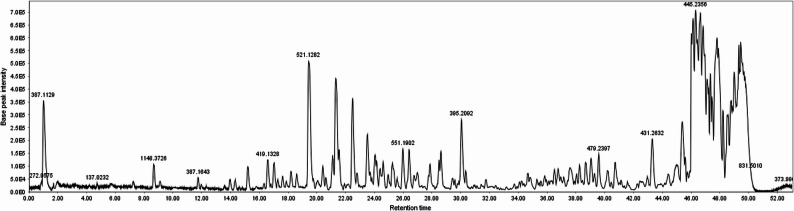
UPLC‒q-TOF‒ESI‒MS/MS chromatogram of metabolites detected in the DCM fraction of *Salvia hispanica* seeds (negative mode)

The bioactive DCM fraction of CH seed was analyzed via UPLC‒q-TOF‒ESI‒MS/MS. A representative UV chromatogram is shown in Fig. 12. The secondary metabolites were identified via SIRIUS software (5.8.6), at a confidence limit of 80%, with the aid of coherency between deprotonated molecular ions and corresponding fragmentation patterns, and by comparison with literature data, as reported in Table 1.

**Table 1:** Peaks of metabolites found in the hydroalcoholic extract of *Salvia Hispanica* L. via UPLC-q-TOF-ESI-MS/MS

Peak No.	compound name	RT	Experimental MWT	molecular formula	fragments	Mean error (m/z) (ppm)	peak area %	Class	Literature
CH1	Sesamose^a^	0.95	665.2122	^C^_24_^H^_41_^O^_21_	383.1177(37.43%), 341.1076(28.28%), 221.0661(23.63%), 179.0546(100%), 101.0229(9.65%)	-2.706	0.025	oligosaccharides	[38]
CH2	Raffinose^a^	0.96	503.1634	^C^_18_^H^_31_^O^_16_	233.0653(29.64%), 179.0550(89.88%), 161.0445(17.57%), 221.0655(28.57%)	4.372	0.118	oligosaccharides	[39]
CH3	trehalose	1	341.1075	^C^_12_^H^_21_^O^_11_	179.0550(53.81%), 161.0456(3.15%), 119.0338(44.88%), 101.0233(89.59%), 143.0344(2.95%), 149.0445(1.93%), 131.0335(7.12%), 113.0233(100%)	-2.638	0.565	disaccharide	[40, 41]
CH4	hydroxyhydrocaffeic acid^a^	2.78	197.0449	^C^_9_^H^_9_^O^_5_	135.0432(100%), 134.0368(63.76%), 123.0454(74.24%), 122.0364(65.50%)	-0.507	0.005	Phenolic acid	[42]
CH5	glucosyringic acid^a^	2.96	359.096	^C^_15_^H^_19_^O^_10_	197.0467(56.90%), 179.0340(100%), 135.0444(60.85%), 123.0439(47.61%)	-5.013	0.012	phenolic glycoside	[43]
CH6	caftaric acid^a^	3.54	311.0397	^C^_13_^H^_11_^O^_9_	179.0331(7.00%), 135.0436(100%), 134.0362(18.09%)	-1.961	0.066	coumaric acids	[42]
CH7	fertaric acid^a^	7.62	325.0559	^C^_14_^H^_13_^O^_9_	193.0493(18.00%), 149.0606(6.35%), 134.0362(100%), 117.0344(3.65%)	-0.185	0.004	coumaric acids	[44]
CH8	glucocaffeic acid^a^	7.66	341.0864	^C^_15_^H^_17_^O^_9_	179.0340(34.38%), 135.0441(100%)	-2.521	0.005	phenolic glycoside	[42]
CH9	osmanthuside H^c^	8.18	431.1571	^C^_19_^H^_27_^O^_11_	191.0556(100%), 149.0444(78.10%), 113.0235(75.62%)	4.082	0.003	Phenylethanoid glycoside	[45]
CH10	ethyl vanillate^a^	10.49	195.0648	^C^_10_^H^_11_^O^_4_	180.0409(18.03%), 136.0154(61.57%), 108.0209(100%), 165.0181(16.89%), 137.0228(34.06%), 109.0299(24.29%), 121.0277(10.44%), 122.0363(8.44%), 107.0148(14.42%)	-4.768	0.016	Phenolic ester	[42]
CH11	Ethylsyringate^a^	10.98	225.0755	^C^_11_^H^_13_^O^_5_	180.0409(14.25%), 165.0182(100%), 137.0231(80.19%), 109.0284(17.01%), 151.0394(2.76%)	-3.554	0.004	Phenolic ester	[42]
CH12	regaloside B	11.03	441.1409	^C^_20_^H^_25_^O^_11_	163.0383(100%),145.0282(84.83%)	2.743	0.002	Phenolic glycoside	[46]
CH13	polygonolide	11.54	219.0653	^C^_12_^H^_11_^O^_4_	232.0369(100%), 135.0073(34.61%), 107.0126(67.26%)	-1.963	0.016	Sesquiterpenoid lactone	Tentative
CH14	metasequirin D	11.61	345.1355	^C^_19_^H^_21_^O^_6_	343.1154(68.64%), 165.0545(100%), 150.0311(99.32%), 149.0228(96.82%), 270.0875(69.55%)	4.868	0.002	Triterpenoid saponin	Tentative
CH15	catechin-o-gallate^a^	11.76	441.0838	^C^_22_^H^_17_^O^_10_	321.0414(34.53%), 279.0315(96.52%)	3.673	0.002	Flavonoid	[47]
CH16	arillatose B^b^	12.95	517.1550	^C^_22_^H^_29_^O^_14_	193.0501(100%), 175.0395(65.41%), 134.0366(57.64%)	-1.431	0.008	Phenolic glycoside	[48]
CH17	lithospermic acid^b^	13.13	537.1012	^C^_27_^H^_21_^O^_12_	339.0479(100%), 295.0601(44.25%), 229.0139(48.94%), 135.0444(13.29%)	-3.928	0.004	Phenolic acid ester	[49]
CH18	methyl homovanillate^b^	14.33	195.065	^C^_10_^H^_11_^O^_4_	150.0306(37.10%), 122.0356(87.33%), 149.0231(100%), 121.0292(77.53%)	-3.742	0.09	Phenolic acid ester	[50]
CH19	Methoxyphlorizin^b^	14.95	449.1431	^C^_22_^H^_25_^O^_10_	165.0545(58.76%), 150.0312(100%)	-3.718	0.007	Flavonoid	[51]
CH20	sinapic acid^a^	15.37	223.0598	^C^_11_^H^_11_^O^_5_	166.0259(94.26%), 148.0152(53.25%), 120.0200(100%)	-3.811	0.004	Coumaric acids	[52]
CH21	Hydroxy-tetramethoxychalcone^b^	16.28	343.1166	^C^_19_^H^_19_^O^_6_	328.0951(20.18%), 298.0841(67.51%), 283.0605(100%), 162.0317(63.58%), 313.0710(66.50%), 297.0771(28.30%), 327.0854(18.53%)	-4.418	0.003	Flavonoid	[53]
CH22	tanegool	16.33	375.143	^C^_20_^H^_23_^O^_7_	327.1230(38.27%), 312.0989(87.49%), 279.0642(100%), 282.0888(53.48%), 267.0654(61.73%)	-3.679	0.132	Lignan	Tentative
CH23	Methoxyphlorizin^b^	16.48	449.1431	^C^_22_^H^_25_^O^_10_	181.0488(100%), 166.0257(47.75%), 135.0442(39.55%)	-3.718	0.009	Flavonoid	[51]
CH24	specioside	16.5	507.1515	^C^_24_^H^_27_^O^_12_	359.0775(14.47%), 323.0762(35.43%), 197.0437(9.12%), 179.0349(31.26%), 161.0231(100%)	2.445	0.004	Iridoid glycoside	Tentative
CH25	Taxiresinol^c^	16.89	345.1336	^C^_19_^H^_21_^O^_6_	267.0654(100%), 109.0286(36.30%)	-0.637	0.003	Lignan	[54]
CH26	ferulic acid^a^	16.95	193.0498	^C^_10_^H^_9_^O^_4_	136.0158(36.60%), 108.0200(48.20%), 133.0286(20.05%), 107.0129(100%)	-1.450	0.011	Coumaric acids	[42]
CH27	methyl homovanillate	17.03	195.0651	^C^_10_^H^_11_^O^_4_	150.0312(100%), 121.0282(55.11%)	-3.230	0.009	Phenolic ester	[50]
CH28	calceolarioside A	17.74	477.1382	^C^_23_^H^_25_^O^_11_	179.0332(34.94%), 161.0232(100%), 133.0280(15.77%), 135.0448(10.38%)	-3.123	0.013	Phenolic glycoside	[55]
CH29	hesperetin dihydrochalcone-o-glucoside^b^	17.83	465.1381	^C^_22_^H^_25_^O^_11_	223.0594(35.16%), 165.0541(100%), 150.0309(55.17%), 181.0489(27.82%), 208.0372(40.81%)	-3.418	0.003	Flavonoid glycoside	[56]
CH30	Salvianolic acid B^b^	18.02	717.1434	^C^_36_^H^_29_^O^_16_	519.0912(24.42%), 475.1030(100%), 353.0650(25.84%), 365.0648(36.35%), 339.0503(53.31%), 321.0425(4.65%), 295.0613(5.69%), 243.0277(13.73%), 229.0124(4.50%), 197.0449(11.50%), 109.0280(4.65%)	-3.026	0.028	Phenolic glycoside	[57]
CH31	fisetin trimethyl ether^b^	18.57	327.0863	^C^_18_^H^_15_^O^_6_	297.0403(37.98%), 253.0508(29.17%), 189.0181(64.95%), 176.0122(43.67%), 162.0319(42.94%), 108.0201(48.81%), 149.0233(26.24%)	-1.712	0.022	Flavonoid	[58]
CH32	ferulic acid^a^	18.66	193.0494	^C^_10_^H^_9_^O^_4_	161.0230(11.17%), 133.0283(100%), 115.0180(0.90%), 105.0332(2.21%)	-3.522	0.095	coumaric acids	[42]
CH33	Salviaflaside^a^	19.43	521.1282	^C^_24_^H^_25_^O^_13_	359.0757(6.71%), 341.0861(3.34%), 323.0757(45.39%), 197.0443(21.38%), 179.0337(28.43%), 161.0232(100%), 160.0154(0.84%), 133.0282(3.60%), 135.0439(11.28%), 117.0334(0.56%), 123.0440(0.97%)	-2.533	1.513	Phenolic glycoside	[42]
CH34	calceolarioside A	19.46	477.1377	^C^_23_^H^_25_^O^_11_	221.0438(3.17%), 179.0346(5.10%), 161.0234(100%), 133.0284(16.15%), 135.0441(11.29%)	-4.171	0.007	Phenolic glycoside	[55]
CH35	caffeic acid 3-glucoside^a^	19.46	341.0866	^C^_15_^H^_17_^O^_9_	179.0345(40.83%), 135.0439(100%), 107.0496(6.13%)	-1.935	0.006	Phenolic glycoside	[42]
CH36	Trimethoxyflavone^b^	19.63	311.0913	^C^_18_^H^_15_^O^_5_	296.0685(37.43%), 251.0708(18.82%), 175.0390(22.82%)	-2.089	0.003	Flavonoid	[59]
CH37	dihydrodehydroconiferyl alcohol 9-o-glucoside	19.74	521.2027	^C^_26_^H^_33_^O^_11_	341.1375(100%), 311.1285(25.04%), 119.0338(56.65%)	0.787	0.026	Lignan glycoside	[60]
CH38	Simplexoside	19.77	517.1714	^C^_26_^H^_29_^O^_11_	337.1077(100%), 322.0818(64.84%), 325.1054(92.24%)	0.793	0.002	Phenolic glycoside	Tentative
CH39	okanin tetramethyl ether^b^	20.24	343.1167	^C^_19_^H^_19_^O^_6_	283.0610(22.22%), 151.0386(43.68%), 136.0153(100%)	-4.255	0.02	Flavonoid	[53]
CH40	rosmarinic acid^a^	21.33	359.0754	^C^_18_^H^_15_^O^_8_	197.0441(7.26%), 179.0334(23.37%), 161.0235(100%), 133.0279(40.86%), 135.0442(31.37%), 123.0453(11.21%)	-3.620	1.303	Coumaric acids	[42]
CH41	Salvianolic acid B^b^	21.35	717.1443	^C^_36_^H^_29_^O^_16_	359.0754(48.91%), 313.0724(9.30%), 197.0440(21.42%), 179.0351(12.72%), 135.0439(7.37%), 177.0175(11.59%), 161.0228(100%), 151.0400(8.41%), 133.0302(7.45%)	-1.813	0.01	Phenolic glycoside	[57]
CH42	Populin	21.55	389.1236	^C^_20_^H^_21_^O^_8_	167.0334(100%), 123.0441(68.81%), 108.0202(18.87%)	-0.128	0.035	Phenolic glycoside	Tentative
CH43	arctignan A	22.23	553.2101	^C^_30_^H^_33_^O^_10_	505.1823(47.85%), 487.1747(34.04%), 475.1739(69.28%), 151.0388(100%)	4.935	0.011	Lignan	Tentative
CH44	naringenin dimethyl ether^a^	22.35	299.0921	^C^_17_^H^_15_^O^_5_	300.0626(100%), 269.0449(37.81%)	0.502	0.028	Flavonoid	[52]
CH45	phillygenin	22.39	371.1506	^C^_21_^H^_23_^O^_6_	165.0556(80.86%), 150.0320(100%)	3.045	0.004	Lignan	Tentative
CH46	imperanene	22.48	329.1404	^C^_19_^H^_21_^O^_5_	177.0544(87.75%), 193.0493(77.10%), 178.0258(100%), 150.0311(58.34%)	4.557	0.04	Flavonoid	Tentative
CH47	Lariciresinol^b^	22.77	359.1512	^C^_20_^H^_23_^O^_6_	193.0494(100%), 178.0258(86.15%), 150.0311(43.05%), 177.0543(64.60%)	4.817	0.008	Lignan	[61]
CH48	Narirutin^b^	22.79	579.171	^C^_27_^H^_31_^O^_14_	353.1012(100%), 338.0775(43.53%), 322.0826(15.23%)	-0.656	0.004	Flavonoid glycoside	[62]
CH49	dehydrodiconiferyl alcohol	22.81	339.1219	^C^_20_^H^_19_^O^_5_	309.0753(100%), 281.0807(48.86%), 263.0703(24.21%)	-3.981	0.05	Lignan	[60]
CH50	Simplexoside	23.01	517.1729	^C^_26_^H^_29_^O^_11_	337.1070(100%), 322.0838(66.81%), 306.0877(34.86%)	3.693	0.107	Phenolic glycoside	Tentative
CH51	terphenyllin	23.25	337.1068	^C^_20_^H^_17_^O^_5_	307.0592(97.60%), 279.0660(100%), 251.0695(12.13%), 291.0635(29.29%), 235.0751(16.53%)	-2.373	0.007	Lignan	[63]
CH52	Luteolin rutinoside^a^	23.36	593.1515	^C^_27_^H^_29_^O^_15_	285.0387(100%), 284.0310(39.95%), 165.0555(88.04%)	1.433	0.003	Flavonoid	[42]
CH53	Phenylapigenin^a^	23.82	337.1061	^C^_20_^H^_17_^O^_5_	219.0801(59.49%), 141.0696(100%)	-4.450	0.022	Flavonoid	[42]
CH54	Lyoniresinol^c^	24.02	419.1688	^C^_22_^H^_27_^O^_8_	237.0766(19.53%), 181.0858(82.97%), 166.0621(100%), 222.0518(30.80%), 192.0419(16.19%), 164.0465(7.77%)	-4.270	0.004	Lignan	[64]
CH55	dihydroxy-trimethoxychalcone^b^	24.13	329.1027	^C^_18_^H^_17_^O^_6_	254.0575(5.33%), 253.0506(6.25%), 149.0594(35.18%), 134.0365(100%), 226.0632(3.35%), 160.0150(19.71%), 148.0152(13.81%), 210.0694(3.89%)	0.547	0.009	Chalcone	[58]
CH56	clinopodic acid A^c^	24.29	343.0805	^C^_18_^H^_15_^O^_7_	179.0342(21.47%), 135.0437(100%), 117.0335(38.02%), 109.0286(3.85%), 145.0282(55.13%), 123.0435(20.36%)	-3.731	0.011	Phenolic acid	[65]
CH57	vaccinoside	24.4	535.1437	^C^_25_^H^_27_^O^_13_	373.0908(42.13%), 179.0339(100%), 135.0439(35.68%)	-2.747	0.033	Phenolic glycoside	Tentative
CH58	acetyl rhein	25.12	325.0339	^C^_17_^H^_9_^O^_7_	281.0469(100%), 237.0570(66.67%)	-2.861	0.105	Anthraquinone	[66]
CH59	coniferyl ferulate	25.26	355.1172	^C^_20_^H^_19_^O^_6_	190.0622(45.64%), 175.0387(100%), 307.0597(40.15%), 295.0595(45.79%), 176.0467(64.00%)	-2.731	0.284	Phenolic ester	[67]
CH60	di-o-sinapoyl glucose^a^	25.26	591.1691	^C^_28_^H^_31_^O^_14_	299.0899(58.64%), 223.0600(100%), 205.0492(52.71%)	-3.857	0.017	Phenolic glycoside	[52]
CH61	clinopodic acid B^b^	25.28	373.0911	^C^_19_^H^_17_^O^_8_	175.0387(75%), 160.0153(48.78%), 179.0338(27.34%), 135.0439(100%)	-3.350	0.063	Phenolic acid	[68]
CH62	catechin-o-gallate^a^	25.3	441.0829	^C^_22_^H^_17_^O^_10_	193.0506(7.65%), 135.0437(100%), 178.0269(7.54%)	1.632	0.006	Flavonoid	[47]
CH63	Fisetin trimethyl ether^b^	25.76	327.0856	^C^_18_^H^_15_^O^_6_	311.0558(75.91%), 283.0597(95.88%), 269.0449(100%), 253.0491(84.63%)	-3.852	0.004	Flavonoid	[58]
CH64	Hydroxy dimethoxychalcone^b^	26.03	283.0980	^C^_17_^H^_15_^O^_4_	314.0775(100%), 283.0590(56.63%), 151.0022(89.72%), 135.0075(58.38%), 107.0126(34.77%), 123.0080(49.27%)	3.532	0.003	Flavonoid	[53]
CH65	Simplexoside	26.07	517.1718	^C^_26_^H^_29_^O^_11_	355.1167(100%), 340.0931(16.72%)	1.566	0.013	Lignan glycoside	Tentative
CH66	kievitone	26.22	355.1171	^C^_20_^H^_19_^O^_6_	311.1261(75.64%), 147.0809(33.08%), 149.0591(58.97%), 135.0435(57.95%), 109.0281(100%), 133.0286(91.79%), 108.0210(49.10%)	-3.013	0.036	Flavonoid	Tentative
CH67	Hydroxy pentamethoxyflavone^b^	29	387.1073	^C^_20_^H^_19_^O^_8_	312.0633(45.71%), 297.0396(100%), 268.0723(59.29%), 253.0486(53.39%), 256.0727(43.93%), 241.0496(37.68%)	-1.808	0.004	Flavonoid	[69]
CH68	3-hydroxy-4”-methoxy-6-methylflavone	29	281.0824	^C^_17_^H^_13_^O^_4_	297.0388(75.79%), 269.0441(45.59%), 253.0499(73.75%), 225.0551(100%), 197.0589(20.72%)	3.593	0.003	Flavonoid	Tentative
CH69	Jaceosidin^a^	32.19	329.0655	^C^_17_^H^_13_^O^_7_	313.0334(331.73%), 299.0182(100%), 271.0237(13.90%)	-1.915	0.023	Flavonoid	[42]
CH70	Gingerol	39.34	293.1743	^C^_17_^H^_25_^O^_4_	221.1533(100%), 236.1044(9.07%), 193.1603(1.69%)	-3.377	0.044	Phenolic	Tentative
CH71	kaempferol tetraacetate^a^	39.42	453.0821	^C^_23_^H^_17_^O^_10_	305.0828(100%), 281.0450(94.95%)	-0.177	0.013	Flavonoid	[42]
CH72	rotundic acid^c^	40.2	501.3197	^C^_30_^H^_45_^O^_6_	471.3097(7.73%), 439.3214(0.76%), 409.3084(5.74%)	-3.830	0.013	Triterpenoid	[70]
CH73	sucrose-laurate	43.62	523.2752	^C^_24_^H^_43_^O^_12_	326.2438(25.15%), 295.2251(100%), 224.1760(41.12%)	-0.478	0.006	Acylated disaccharide	Tentative
CH74	rotundic acid^c^	45.54	487.3405	^C^_30_^H^_47_^O^_5_	469.3309(10.07%), 437.3036(1.68%), 393.3140(2.39%), 443.3526(0.88%)	-3.796	0.051	Triterpenoid	[70]
CH75	asiatic acid^b^	45.96	487.3407	^C^_30_^H^_47_^O^_5_	469.3279(0.53%), 409.3107(1.42%)	-3.386	0.036	Triterpenoid	[71]

^a^reported in *Salvia hispanica*

^b^reported in *Salvia*

^c^reported in Lamiaceae

UPLC‒q-TOF‒ESI‒MS/MS metabolome profiling approach revealed the presence of a wide array of phenolic compounds, such as phenolic acids, flavonoids and coumaric acids.

*CH3* represented trehalose with deprotonated peak at *m/z* 341.1075 and peak area of 0.565%. It displayed MS^2^ ions at *m/z* 161.0456 due to the cleavage of glycosidic linkage between the two sugar units followed by dehydration (-18 Da) that formed a fragment ion at *m/z* 143.0344 [40].

*CH5* was identified as glucosyringic acid that exhibited [M − H]⁻at *m/z* 359.096. The fragmentation of the parent ion showed a base peak at *m/z* 179.0340, that resulted from subsequent dehydration and molecular ion peak at *m/z* 197.0467 due to deglycosylation [43].

*CH6* was caftaric acid with [M − H]⁻at *m/z* 311.0397 with a product ion at *m/z* 179.0331 [M − H−132]⁻due to the presence of deprotonated caffeic acid after ester linkage cleavage. A base peak was demonstrated at *m/z* 135.0436, characteristic to decarboxylation of the parent ion [42].

Fertaric acid (*CH7*) demonstrated a precursor ion peak [M − H]⁻at *m/z* 325.0559 where the typical fragmentation pattern of tartaric acid was exhibited as: fragment anion at *m/z* 193.0493 owing to the loss of ester bond and thus corresponding to ferulic acid moeity. Product ion at *m/z* 149.0606 was tentatively identified as a product of the decarboxylation of deprotonated parent peak. Further loss of CH_3_ group (− 15 Da) led to formation of fragment ion at *m/z* 134.0362 [44].

Methyl homovanillate (*CH27*) of deprotonated molecular ion that is depicted at *m/z* 195.0650, besides a base peak shown at *m/z* 149.0231 due to ester bond breakage followed by demethylation. In addition, a prominent fragment ion was detected at *m/z* 121.0292 resulting from the decarboxylation (− 28 Da) [50].

Peak *CH32* was assigned to ferulic acid with [M − H]⁻at *m/z* 193.0494. It exhibited a fragment ion at *m/z* 133.0286 that resulted from decarboxylation and demethylation, with the reported base peak that appeared at *m/z* 107.0129 [42].

Peak *CH33* demonstrated the anion [M − H]⁻ at *m/z* 521.1282 which postulated the identity of salviaflaside (rosmarinyl glucoside), that is confirmed by the classical fragmentation of rosmanoyl derivatives such as molecular ion at *m/z* 359.0757, which resulted from sugar moiety loss (− 162 Da), and *m/z* 353.0757 from danshensu group loss (− 198 Da). A base peak appeared based on dehydration of caffeic acid at *m/z* 161.0232. Further fragmentation yielded ions at *m/z* 197.0443 and *m/z* 179.0337 which are consistent to anions of hydrocaffeic acid and caffeic acid, respectively [42].

Rosmarinic acid (*CH40*) showed [M − H]⁻at *m/z* 359.0754 with recognizable peak area of 1.303%, that showed a fragment at *m/z* 197.0141 due to breakage of the ester bond between caffeic acid and danshensu, while the loss of two water molecules formed the base peak at *m/z* 161.0235 [42].

*CH58* was identified as acetyl rhein of [M − H]⁻at *m/z* 325.0339. Its MS/MS spectrum showed a base peak at *m/z* 283.0272 due to deacetylation, followed by decarboxylation that led to formation of a product ion at *m/z* 239.0375 [66].

*CH59* was assigned as coniferyl ferulate of [M − H]⁻at *m/z* 355. The fragmentation yielded *m/z* 193.0622 which is in agreement with the reported data of feruloyl moiety, while the base peak at *m/z* 178.0387 resulted from demethylation of the base peak [67].

Clinopodic acid B (*CH61*) showed molecular anion at *m/z* 373.0911. Ester linkage cleavage followed by dehydration produced fragment ion at *m/z* 179.0341, which was then decarboxylated to form base peak at *m/z* 135.0439 [68].

## Discussion

Nature has always offered an unlimited supply of bioactive compounds and drugs for humanity with huge benefits towards combating complex health issues. It provides a wide range of chemical scaffolds and moieties in the form of natural products such as plants and microorganisms. Unlike primary metabolites, plants are able to synthesize and store many secondary metabolites that are not fundamental for cellular metabolism. These secondary metabolites are usually called phytochemicals or natural products. They have a wide array of chemical structures, enabling them to act on different molecular targets and receptors, resulting in various pharmacological activities [72].

Several edible plants, under the umbrella of functional foods or nutraceuticals, have shown promising antioxidant, anti-inflammatory and neuroprotective activities [73]. They contain secondary metabolites such as phenolics, including flavonoids, isoflavonoids and lignins, which are potent radical scavengers and powerful antioxidants comparable to well-known antioxidants such as vitamin C. These compounds are already used for human consumption, making their application as nutraceuticals simpler than their use as medicinal plants [72].

There is a harmful impact of free radicals and they have a deleterious role in a wide range of human illnesses. Therefore, food containing antioxidants such as spices, vegetables and fruits has been shown to hinder the process of aging and decrease the incidence of various genetic disorders [74–76].

UPLC-q-TOF-ESI-MS/MS revealed several peaks indicating several compounds of significant importance that contribute to the activity of the DCM fraction of CH seed. The DCM fraction was chosen based on its distinctive profile of intermediate-polarity phytoconstituents relative to the other fractions. Caffeic acid, and its derivatives, are believed to be major contributors to the activity of CH seed. They have previously reported antioxidant and neuroprotective effects against neurotoxicity induced via β-amyloid [77]. Caffeic acid phenethylester has previously increased the nuclear translocation of the DAF-16 transcription factor in *C. elegans*, stress resistance and lifespan [78]. Additionally, rosmarinic acid derivatives were observed. Rosmarinic acid, which was previously examined in *C. elegans*, improved oxidative stress and decreased the degree of neuronal damage caused by α-synuclein and polyglutamine [79]. Furthermore, salvianolic acid was detected in our fraction. It previously could upregulate the expression of several antioxidant and detoxification genes in the *C. elegans* as *gst-4*, *gst-10*, *spr-1*, and *trxr-2* [80]. Ferulic acid, also detected in the DCM fraction, could previously extend lifespan and improve healthspan in the *C. elegans*. It enhanced locomotion, reduced fat and polyglutamine aggregation, and improved resistance to heat and oxidative stress by lowering ROS levels. These effects were mediated through upregulation of *hlh-30*, *skn-1*, and *hsf-1* genes, which are dependent on insulin/IGF-1 signaling pathway [81]. Fertaric acid, a hydroxycinnamic acid derivative with promising antioxidant activity, was also found in the DCM fraction [82]. Caftaric acid, also a hydroxycinnamic acid derivative, was present in the DCM fraction. It has previously shown anti-inflammatory and antioxidant effects in rats [83]. Moreover, specioside, an iridoid glucoside, which prolongs the life span and ameliorates oxidative stress in *C. elegans* worms was detected in the DCM fraction [84]. Several flavonoids that were previously reported to have antioxidant effects, such as a kaempferol derivative, were also detected in the DCM fraction. Kaempferol could contribute to the total activity of CH seed, as it has already shown potent antioxidant and neuroprotective activity in disorders such as Parkinson’s disease [85, 86]. It was tested in a *C. elegans* worm model, where it increased survival, lowered intracellular ROS levels and increased the nuclear localization of DAF-16 [87]. The fraction is enriched with many other flavonoids with reported antioxidant activities such as jaceosidin flavone and fisetin flavonol [88, 89]. Lignans, with antioxidant and neuroprotective effects, were also detected such as lyoniresinol [90]. Sugars like trehalose, present in our fraction, was previously studied and reported as a stress protectant in *C. elegans*. It could extend the mean lifespan of worms by over 30% in case of young adult stage. When administered to older adults, it could prolong remaining lifespan by 60%. It was able to result in reduced mortality, prolonged reproductive span, preserved pharyngeal pumping activity, decreased lipofuscin accumulation, enhanced thermotolerance and reduced polyglutamine aggregation.

Limited literature discussed the neuroprotective effects of chia seed. Thus, it was interesting to explore its effects on the development of HD in this study. Here, we demonstrated that CH seed could significantly decrease polyQ150 aggregation in ASH neurons, leading to protection from neuronal death and enhancing the chemotaxis ability of worms. The results also revealed a decrease in polyQ35 and polyQ40 aggregates in the muscles of the worms, leading to increased motility.

Phenolics, found within the DCM fraction, are thought to be responsible for the antioxidant effect and might potentially participate in prevention of improper misfolding or aggregation of polyQ. This might be due to their ability to create a spacer effect, preventing neurotoxic protein assembly [91]. Phenolics can bind through noncovalent, covalent and ionic bonds, in addition to hydrophobic interactions, to proteins such as huntingtin, resulting in the inhibition of oligomer and subsequent aggregate formation [92].

The observed neuroprotective activity against HD-associated phenotypes could be also linked to the antioxidant effect of CH seed, as it improved the oxidative stress resistance of the worms in the survival assay after exposure to juglone in both N2 and CF1038 strains. This result demonstrates that the oxidative stress resistance activity of CH seed could involve other pathways like Ld-1 SKN-1 and might not be strictly dependent on the activation of DAF-16 pathway. CH seed also has good antioxidant potential through scavenging ROS and decreasing their levels inside worms. It was able to activate the DAF-16/FOXO pathway, a pathway responsible for controlling lifespan and genes affecting metabolism and response to stress, and increase its nuclear localization [93]. Moreover, the involvement of the DAF-16 pathway was further supported by increased SOD-3 expression and decreased HSP-16.2 expression after exposure to a non-toxic dose of juglone. This effect was previously reported for various plant extracts and polyphenolic compounds with antioxidant activity [28, 29, 94]. Additionally, it is believed that activation of the DAF-16/FOXO pathway is linked to neuroprotection through neuronal compensation in diseases such as HD. A former study documented that compounds like (PhSe)₂ demonstrated significant neuroprotective effects in *C. elegans* models of HD, where it reduced polyglutamine aggregation, prevented neuronal degeneration, and preserved sensory neuron function. In addition, it decreased ROS levels and improved both lifespan and health span in worms. These effects were likely mediated through modulation of the insulin/DAF-16 signaling pathway and activation of downstream protective genes, including *hsp-16.2* and *sod-3* [95]. Previous studies also have demonstrated that neuroprotective compounds can act via DAF-16/FOXO–dependent pathways, which are crucial for maintaining neuronal resilience in HD models. For example, neurosteroids (e.g., MAP4343, 17β-estradiol), flavonoids (e.g., resveratrol and isoquercitrin), and GSK-3β inhibitors (e.g., LiCl) have been shown to require DAF-16/FOXO activity to sustain neuronal function in *C. elegans* expressing polyglutamine-expanded huntingtin. These compounds modulate signaling cascades involving SIRT1, AMPK, and β-catenin, which converge on FOXO to regulate stress response and cellular homeostasis. Therefore, enhancing FOXO signaling could represent a key mechanism underlying the observed neuroprotective and stress-resistant effects of CH seed [96].

## Conclusions

The present study sheds light on the neuroprotective potential of CH seed DCM fraction against HD-associated phenotypes using the *C. elegans* worm model. By decreasing ROS levels, activating the DAF-16/FOXO pathway and its downstream target genes, *sod-3* and *hsp-16.2*, CH seed has demonstrated antioxidant capacities. Through enhancing the survival of worms stressed by juglone, oxidative stress resistance activities were demonstrated. We also infer that CH seed is capable of protecting against HD-associated phenotypes by reducing the accumulation of the following aggregates: polyQ35, polyQ40, and polyQ150. The involvement of the DAF-16 pathway might be related to anti-HD activity. However, it is impossible to exclude other unknown mechanisms. For future studies, the involvement of the Ld-1 SKN-1 pathway should be tested. To elucidate the specific molecular pathways influencing CH seed activity, more research is still needed using higher mammalian model organisms.

## Data Availability

Data of this study are present upon request from the corresponding author.

## Abbreviations

ASH: Amphid sensilla
DCM: Dichloromethane
*C. elegans*: *Caenorhabditis elegans*
Chia: CH
ESI: Electrospray ionization
*E. coli*: *Escherichia coli*
FUDR: 5′-fluorodeoxyuridine
FOXO: Human fork head transcription factor
GFP: Green fluorescent protein
HD: Huntington’s disease
Htn: Huntingtin
NGM: Nematode growth medium
PolyQ40: Polyglutamine
ROS: Reactive oxygen species
SOD-3: Superoxide dismutase 3
